# Prostatic Hypertrophy Treatment1See p. 715, July, 1906, issue of this Journal.

**Published:** 1906-09

**Authors:** E. A. Babler


### SELECTION.

---

### Prostatic Hypertrophy Treatment.[1]

[*St. Louis Courier of Medicine, July, 1906.*]

CONCLUSION.

Previous to the accomplishments of the renowned McGill, of Leeds, the treatment of prostatic obstruction was principally palliative; the medical attendant advocated catheterism in practically all of these cases, since it seemed to yield results far superior to those obtainable by other methods. Today, however, the one important and pressing question presenting itself for prompt decision in every case of prostatic obstruction, is: is the medical attendent justified in introducing this sufferer to catheterism, with all its attendant dangers, distressing, and often fatal, complications, when prompt and proper surgical intervention,—operation before the appearance of septic cystitis, renal infection, renal insufficiency, or other conditions which destroy the patient's health, —yields the safest and the most satisfactory outcome? Personally, I can not help but feel that when the pathologic changes in the prostate have reached such a stage that it is necessary to subject the sufferer to the dangerous and merely palliative treatment (regular catheterisation), certainly the condition fully warrants prompt surgical intervention (radical removal of the diseased tissue); hence, I am sure that Dr. H. G. Mudd is taking a decidedly advanced step in the right direction when he says:

"In my opinion the safe plan in the great majority of the cases would be to remove the offending organ with the first alarm sounded by the symptoms of obstruction."

The concensus of English opinion is tersely expressed by Sir Frederick Treves when he says:

"As long as a patient with enlarged prostate can live in comfort with the occasional use of the soft catheter, no operation is to be advised. But when catheterism becomes increasingly frequent and difficult, the question of radical operation has to be considered. Recent experience has shown that the risk of prostatectomy is much less than it was formerly considered to be; and further, that the complete removal of large adenomata which usually form the obstruction is attended by results with which those following partial excision can not be compared."

---

1. See p. 715, July, 1906, issue of this Journal.

Syms feels that operation is indicated:

1. When the patient suffers from obstruction which necessitates very frequent urination to a degree which entails exhaustion.

2. When he suffers from repeated attacks of acute cystitis, or from a marked degree of chronic cystitis.

3. When bladder stone is caused by prostatic obstruction.

4. When frequently repeated hemorrhages occur.

5. When the pain tends to undermine the patient's health.

A careful study of the pathology and clinical manifestations of prostatic hypertrophy, and of the literature concerning the treatment, clearly demonstrates the fact that the results of palliative treatment are absolutely unsatisfactory—in many instances the dangers accompanying their application far exceeds the dangers attendant upon early prostatectomy. Concerning the chief dangers of radical operation Mudd says:

"The chief danger of prostatectomy now lies in the fact that the condition of the patient coming to operation is such that any surgical interference is a matter of great gravity."

Renal insufficiency is looked upon by many surgeons as a distinct contraindication to radical operation. Concerning some of the contraindications Fuller says: "Putrid urine and the presence of an ascending pyelitis together with some involvement of the kidney, should not stand in the way of operation, but their existence should strengthen the plea for speedy relief of the prostatic obstruction, the direct cause for their presence and combination; radical operation is often followed by perfect recovery."

Such antique and unscientific treatment as ligation of the internal iliac arteries, castration, vasectomy, angioneurectomy, etc., have, thank heaven, sunken into unmoaned and unhonored graves. Robinson sought to perform prostatotomy by way of the rectum, while Harrison performed external perineal urethrotomy, and then inserted the knife into the prostatic urethra, dividing the prostatic bar on the floor of the gland, then he forcibly stretched the prostatic urethra with his fingers or a sound. Mercier devised the prostatome for cutting the middle lobe of the gland through the urethra. Gouley presented an instrument for cutting or punching out segments of the enlarged gland; the instrument was inserted through a perineal incision. Maisonneuve devised an instrument whereby he was enabled to incise the prostate through the floor of the urethra. In 1877 Bottini, of Italy, modified Mercier's instrument in so far as to produce a galvanocaustic incision which burned instead of cut. Freudenberg greatly improved Bottini's instrument. More recently Chetwood has presented a similar instrument which he introduces through a perineal incision. The advantages of the Chetwood instrument are quite obvious; the bladder can be carefully explored with the finger before the instrument is introduced; the operator clearly knows what he is doing; and it is claimed, that the operation is applicable in almost all cases. In former years suprapubic prostatotomy was frequently resorted to; it is a fact that all of these methods are palliative, and have many objectionable features,—in many instances they have been resorted to simply because the patient's condition prevented radical operation.

And now we come to the recently much-discussed and, I think, ideal treatment,—excision of the diseased prostate. Previous to 1886, prostatectomy for the relief of urinary obstruction was an operation that was unknown. Mr. McGill, of Leeds, not only originated the operation, but improved and perfected it. In fact, the medical world looks upon Mr. McGill as the father of advanced prostatic surgery. McGill began by removing small pieces of the prostate in an unscientific way through the bladder after a suprapubic cystotomy. The perfected operation was as follows: After having performed suprapubic cystotomy and sutured the bladder to the abdominal parieties, he then inserted curved scissors through the incision and snipped through the membrane over the projecting portion of the prostate, completing the operation by enucleating the gland with the fingers and forceps. The bladder was drained suprapubically, the bladder having previously been irrigated with water so hot as to make it uncomfortable for the hand. Bellfield modified McGill's technic by advocating the additional operation of perineal urethrotomy to facilitate drainage, while Fuller advised counter-pressure by means of the fist against the perineum. Of late Freyer has reported excellent results with suprapubic prostatectomy—according to an editorial in the November, 1905, issue of the Buffalo *Medical Journal,* the technic employed by Freyer is that advocated by Fuller; in fact, Freyer is severely criticised for failing to credit Fuller with the technic.

To Nicoll belongs the credit of having been the first to devise a well defined perineal operation for removal of the diseased prostate. Nicoll's technic was the following; after performing suprapubic cystotomy and attaching the bladder to skin, an incision was then made in the perineum, similar to that in perineal urethrotomy, and a second one at right angles to it in front of the anus, forming a T. Nicoll then dissected up in the perineum anterior to the rectum as far as the prostate, and having cut through the capsule he inserted his right forefinger between it and the gland; the fingers of the left hand were employed to make counter pressure from above, while enucleation was being performed by the right forefinger. Nicoll drained through the urethra by means of a retained catheter without performing urethrotomy. The lower wound was packed with gauze; the sutures holding the bladder and abdominal wall in apposition were then removed, and the bladder allowed to fall back into the abdomen.

Later, Alexander advocated going into the prostate through a perineal incision made into the membranous urethra up to the apex of the gland; the capsule was then incised and the finger inserted in the latter for enucleation. Counterpressure through the suprapubic cystotomy incision was employed. Both suprapubic and perineal drainage were employed.

Syms attempted to obviate the necessity of suprapubic cystotomy by inventing a rubber retractor. This hard rubber tube with a soft rubber balloon at the end is too well known to require further comment. Recently Syms has reinforced the rubber balloon with a canvas lining. In 34 cases Syms has found it perfectly satisfactory.

More recently, Young has presented an excellent modification of the metal refractor of the French. Young contends that no important structures are injured by his technic, and the entire exposure of the prostate is by incising the skin and dividing the central tendon and the insignificant recto-urethralis muscle beneath it in the median line,—(both of which are necessarily severed in all perineal exposures of the prostate) the remainder of the operation is done by blunt dissection with the forefingers. The levator ani muscles and other important structures are merely separated and held apart by retractors so that no more injury is done than in other perineal operations. Young claims that the entire operation is under the eye of the operator; that the sexual powers will be preserved; that proper attention can be given the urethra and to the ejaculatory ducts,—in a word he feels confident that it is the most simple and most satisfactory technic.

It must be admitted that many surgeons highly commend the Young technic. Mudd feels that the method advocated by Young is the operation of choice; the whole operation can be done quickly and deliberately under the guidance of sight and touch. His objection to the Bryson technic lies in the fact that there must necessarily be wide destruction of the prostatic urethra, and the uncertainty of completeness; the bladder and the rectum are frequently torn into.

Today we find the English clinging to McGill's technic, the Germans lean toward the Bottini-Freudenberg instrument, while in America the perineal technic is constantly gaining ground. Personally, I can not help but feel that no one operation is suitable for all cases, so much depends upon the site and character of the obstruction, and the general condition of the patient. Just as soon as the medical attendant fully appreciates the fact that the distressing results of palliative treatment can be prevented by a more safe procedure,—one that yields satisfactory results,—just so soon will we see the best results of prostatectomy.—E. A. Babler.

